# Kuwaiti women’s perception of social support in pregnancy: a qualitative study

**DOI:** 10.1186/s12884-025-08465-4

**Published:** 2026-04-16

**Authors:** Mona Al-Mutawtah, Mihela Erjavec, Hans-Peter Kubis, Emma Campbell

**Affiliations:** 1 School of Psychology and Sport Sciences, Bangor University, Bangor, UK; 2 Community Medicine- Clinical Psychology, Kuwait University, Kuwait City, Kuwait

**Keywords:** Pregnancy, Social support, Qualitative research, Reflexive thematic analysis, Traditional gender roles, Traditional family roles, Cultural beliefs, Marital relationship

## Abstract

**Background:**

Pregnancy is a significant event in a woman’s life. However, it also presents challenges due to physiological and psychological stress and changes in appearance, roles, and lifestyle. Lack of social support is recognized as a risk factor affecting pregnant women’s well-being. In this study, we aim to investigate Kuwaiti women’s perceptions of social support (emotional, instrumental, and informational) during pregnancy and lifestyle changes and whether cultural factors influence their perception.

**Methods:**

This cross-sectional qualitative study of 31 Kuwaiti pregnant women utilized a constructivist approach to emphasize participants’ active role in knowledge construction, acknowledging the influence that social and cultural factors have on their narratives. We chose reflexive thematic analysis (RTA) due to its capacity to offer comprehensive analysis and reveal hidden themes. The semi-structured interview collected data on lifestyle changes and the types of social support women experienced during pregnancy (emotional, instrumental, and informational).

**Results:**

RTA revealed three main themes and two subthemes. The first theme, ‘The Female Community as a Double-Edged Sword,’ encompasses two subthemes: the sense of belonging and connection and the pressure to be perfect, in connection with Kuwaiti social norms. The second theme highlights the impact of traditional gender and family roles on women’s pregnancy experiences, limiting support. The third theme focuses on the importance of the marital relationship in providing stability, security, love, and support during pregnancy. However, many women expressed dissatisfaction with their husbands’ lack of understanding and emotional support during pregnancy.

**Conclusions:**

This research fills gaps in the existing literature, providing valuable cultural insights and advocating for context-specific support measures in the Gulf region, paving the way for further exploration in this field.

**Supplementary Information:**

The online version contains supplementary material available at 10.1186/s12884-025-08465-4.

## Background

Pregnancy is a unique and natural event in a woman’s life. However, it also presents challenging for many women, as it involves significant physiological and psychological stress, changes in appearance, role, and lifestyle [1, 2], often resulting in poor emotional well-being. According to the World Health Organization [3], mental illness affects 10% of pregnant women and 13% of postpartum women worldwide. Moreover, it is estimated that more than 20% of pregnant women experience high levels of anxiety during their first trimester, and more than 9.5% suffer from depression during that period [4]. High stress levels are associated with a wide range of adverse health outcomes for mothers, including depression, panic disorder and substance use [5–7]. Insufficient social support is a key risk factors impacting the well-being of women during and after pregnancy [8, 9].

Social support refers to the assistance and encouragement provided by a person’s social network, which helps individuals cope with stress, mange tasks, and achieve personal goals [10, 11]. In the mental health literature, social support is conceptualised as interpersonal interaction that consists of emotional (affection, love, comfort, reassurance), informational (guidance, advice, skills), and instrumental (tangible help such as money, goods or services) components [12–16]. Good social support has a positive impact on physical health and psychological well-being [17]. Studies have shown that it plays an essential role in reducing stress during pregnancy, improving coping skills, preventing depression, and enhancing overall well-being [18–22]. Harrison et al. [23] found that low levels of social support during pregnancy and the postpartum period have been associated with an increased risk of pre-and postnatal depression and anxiety symptoms. Support from the spouse, family members, friends, and healthcare professionals acts as a buffer against stress and improves overall health and well-being [12, 24]. Specifically, effective emotional and instrumental support appears to mitigate the negative effects of life stress on the mother’s emotional well-being [9, 25, 26].

Cultural differences play a significant role in how social support is perceived and provided. In European American cultures, emotional support (e.g., encouragement and reassurance) is more prevalent and is associated with improved psychological outcomes. In contrast, instrumental support (e.g., tangible aid) is more commonly provided in Asian American culture [13]. Cultural psychology research has revealed that the social norms governing relationships vary significantly among cultures. For instance, in more individualistic cultures, such as those in North America and Western Europe, people are encouraged to assert their independence and distinctiveness. By contrast, more collectivist cultures, including East Asia and Arab societies, emphasise group harmony and interdependence. Individuals in these cultures may be more hesitant to seek help, concerned about disrupting social harmony, receiving criticism, losing face, or burdening others — believing instead in managing their own problems independently [11, 27–29]. In Arab cultures, social norms are deeply rooted in traditional religious values, such as showing respect and politeness, particularly to the elderly. These values may also influence how and when individuals seek social support. Many Arabs believe their fates are predetermined, which may reduce the inclination to seek help proactively [30–33]. Arab societies also prioritise relational ties over individual achievement, further shaping patterns of social support [34]. Due to this, an individual is more likely to stand back in their demands rather than risk friction with social norms and group harmony.

Kuwait is a small Muslim nation in the North of the Arabian Gulf with a local population of about 1.45 million citizens, and a comparably wealthy country with a very high per capita income for its citizens [35]. The root of the population is mostly from Najd (Saudi Arabia), Iraq, and Iran, which means that the majority are Bedouin. Most Kuwaiti citizens live in highly developed cities, with high living standards and access to higher education [36].

Due to strong interactions with Western societies, based on technology, media, education and trade, family life is diverse, participating in both Western culture and traditional Kuwaiti family lifestyles [37]. Many Gulf cultures, including Kuwait, do not expect men to assist with household duties or child-rearing, which can limit the support available to women [38]. Reflecting these traditional gender roles, Kuwaiti society generally places men in charge of household matters, while women handle all childcare and household duties (see James-Hawkins et al. [39] for a parallel with Qatar) [40]. Additionally, traditional family roles in Kuwaiti societies play a major role and may allow in-laws within Kuwaiti families to express critical views about a woman’s pregnancy or motherhood choices, which may lead to intrusive or controlling behaviour.

In recent decades, there has been a shift toward smaller family sizes in Kuwait. World Bank [41] data indicates that Kuwait recorded 2 births per woman in 2021, which represents a significant decline compared to historical data, where, for instance, the fertility rate stood at 7.1 in 1961.

Despite the fact that social support seems to play a fundamental role in promoting women’s well-being during pregnancy, there is a lack of research in this field in the Arab region. In addition, recent studies are not considering whether culture-specific factors may influence how women perceive social support in Kuwait. Therefore, the present research aimed to explore how Kuwaiti women perceive social (emotional, instrumental, and informational) support during pregnancy and lifestyle changes, and whether culture-specific factors influence their perceptions.

## Methodology

### Design

This is a cross-sectional qualitative study, in which participants’ experiences were captured at a single point in time through semi-structured interviews with a convenience sample of married Kuwaiti pregnant women, without age or health restrictions. Given the novelty of the research aim, a qualitative approach was selected as it provides insight into individuals’ perception of social support within their specific culture context—something that quantitative approaches (e.g., questionnaires) developed in Western cultures may not fully capture [42]. Reflexive thematic analysis (RTA) was therefore chosen as the most appropriate method for comprehensively exploring participants’ perceptions, experiences, needs, views, and meaning-making regarding the issues under investigation, following the guidelines of Braun and Clarke [43]. In addition to providing insight into participants’ lived experiences, this method acknowledges the importance of subjectivity and reflexivity throughout the research process [42].

A constructivist perspective was adopted, as it highlights how knowledge is actively constructed and understood by participants. This approach values diverse and subjective experiences while recognising the influence of social and cultural factors in shaping participants’ narratives [44]. By foregrounding the complexity and contextual specificity of pregnant women’s realities in Kuwait, this perspective offers a deeper understanding of their experiences. The study adhered to the Standards for Reporting Qualitative Research (SRQR) guidelines [45].

### Ethics

The study protocol was approved by the Bangor University Ethics and Governance Committee (application number 2021–16885-A14808) and the Kuwait Health Authorities (application number 2021/1689). Participation was entirely voluntary, and participants were informed of their right to withdraw at any time without providing a reason. Participants’ anonymity was assured, and they were fully briefed about the study’s purpose, with opportunities to ask questions. Written informed consent was obtained for participation and the use of anonymised quotes in the research report.

Following each interview, a debriefing letter was sent summarising the key aspects of the study. It also provided contact details for the research team in case participants had further questions or concerns, and included information about relevant support services, acknowledging that discussions about pregnancy and mental health might evoke emotional responses. As a culturally appropriate gesture of appreciation, participants received a foot massage gift voucher. A recent study indicates that modest incentives effectively acknowledge participants’ contributions without compromising the voluntariness of their participation [46].

### Sample size

In reflexive thematic analysis, the sample size is guided by the aim of generating rich, nuanced, and meaningful themes rather than achieving data saturation. Rooted in an interpretivist epistemology, RTA emphasises depth of engagement with each participant’s account over redundancy of responses. Therefore, the appropriate sample size depends on the analytic scope and complexity of the research. Braun and Clarke [47] suggest that when conducting interviews as part of a large study, a minimum of 20 participants is suitable.

### Participants

Participants were 31 married Kuwaiti pregnant women living with their husbands, with a mean age of 32 years (range: 24–41). The number of children ranged from 0 to 6. Additional demographic information is presented in Table 1.

**Table 1:** The participant characteristics

Characteristics	Number of participants
Education	
Bachelor’s degree	21
College students	2
High school	1
Diplomas	4
Masters	2
PhD	1
Location/Region	
Hawalli Governorate	3
Al Asimah Governorate	8
Mubarak Alkabeer Governorate	7
Ahmadi Governorate	3
Farwaniya Governorate	5
Jahrah Governorate	5
Employment Status	
Employed	20
Medical leave	6
Homemakers	3
Students	2
Month of Pregnancy	
Two	1
Three	3
Four	5
Five	4
Six	5
Seven	4
Eight	7
Nine	2

### Data collection: The semi-structured interview

Semi-structured interviews were selected as the primary method of data collection, focusing on lifestyle changes and the three types of social support (emotional, instrumental, and informational) during pregnancy. Some questions also addressed cultural influences. The interviewer chose to conduct audio interviews via WhatsApp calls, as this method facilitates discussion of sensitive topics and allows participants to disclose essential and often hidden aspects of human behaviour [48].

While previous research has shown that online and telephone-based interviews may reduce access to visual cues, such as body language and facial expressions—potentially affecting rapport and data interpretation [49, 50]—other studies suggest that audio interviews may encourage more open expression, particularly on sensitive topics, due to a heightened sense of privacy [51]. Semi-structured interviews are well suited to exploring individuals’ perceptions and experiences of social support during pregnancy [47, 52]. This method allowed both the researcher and participants to cover predetermined themes while leaving the room for the conversation to develop naturally [53], enabling the co-construction of knowledge throughout the interview process [54].

The interviewer used a semi-structured interview guide that began with introductory questions to help participants ease into the conversation, as recommended for well-designed interview schedules [47]. The guide then proceeded to two sections of open-ended questions focusing on: (a) pregnancy (e.g., ‘Has your pregnancy so far been what you expected?’; ‘Has the pandemic affected your experience of being pregnant?’; ‘Have your lifestyle and daily activities changed?’) and (b) social support (e.g., ‘Who do you talk to about how you are feeling?’; ‘Who do you turn to for practical help and advice or information?’). A list of prompts and probes was also used to clarify (‘Can you please clarify/explain more?’), elaborate (‘Can you tell me more about that?’), or seek more detail (‘Who do you do this with?’). The interview concluded with an open question (e.g., ‘Is there anything else that you would like to share with me today?’) to allow participants to raise topics not previously covered [55]. Interviews lasted between 30 to 108 min, were digitally recorded, transcribed verbatim in Arabic, and translated into English for analyses. Each translation was meticulously reviewed by the first author and the research team to ensure that meaning was preserved and true to the original transcripts.

### Data collection

The first author conducted individual audio interviews between October and December 2021. Participants were recruited via online advertisements on social media and through snowball sampling (where participants or the first author’s colleagues and friends referred other pregnant women). The first author, an experienced clinical interviewer, was skilled at managing distress and creating a comfortable environment. Many participants expressed satisfaction with the interview process, with comments such as: “*Wow, thank you very much. I didn’t feel that this was an interview, and I’m shocked that an hour passed as we were talking. I didn’t feel it.*”.

### Data analysis: Reflexive Thematic Analysis (RTA)

Reflexive Thematic Analysis (RTA) was selected as it is particularly suited to understanding the complexity of participants’ stories [55]. RTA allows for in-depth analysis of qualitative data, helping identify both explicit and implicit themes. This holistic approach enhances the comprehensiveness of participants’ experiences [56]. The aim was to explore each participant’s narrative in detail rather than to produce a uniform or generalised account.

### Data processing

This study followed the six-phase analytical process outlined by Braun and Clarke [43]. The first phase involved repeatedly reading of transcripts to become deeply familiar with the dataset. During this process, researchers actively engaged with the data by making observations, identifying patterns and asking questions.

The second phase involved generating initial codes to organise the data meaningfully. Key data features were highlighted using concise labels. An inductive (data-driven) coding approach was adopted, meaning that cods and themes determined from the data itself, rather than being imposed a priori [57].

In the third phase, related data segments were grouped, and codes were reviewed to identify patterns. Themes and codes were developed at both semantic and latent levels. Semantic coding identifies surface meaning, whereas latent coding captures underlying ideas, assumptions or concepts not explicitly stated in the data [58].

In the fourth phase, the candidate themes were reviewed, and a theme map was developed. During the fifth phase, themes were refined and clearly defined. The first author wrote detailed analyses of each theme, ensuring they captured the essence of the narratives and their relationship to the data overall.

The final phase involved producing the research report, integrating findings with the with the literature in both the results and discussion sections [47, 58, 59]. The first author led the analysis and engaged the rest of the research team—consisting of ME (a psychology researcher), HK (experienced in both quantitative and qualitative human research) and EC (an expert in qualitative health research)—in ongoing discussions. All team members contributed to the analysis by reading transcripts, discussing codes, and collaboratively refining themes, enhancing the reliability and depth of the findings.

In RTA, researchers may choose either an illustrative or analytical approach to presenting themes. An illustrative approach defers connections to the literature until the discussion section, while an analytical approach integrates literature during theme presentation [57, 60]. This study followed an analytical approach.

## Results

**Fig. 1:**
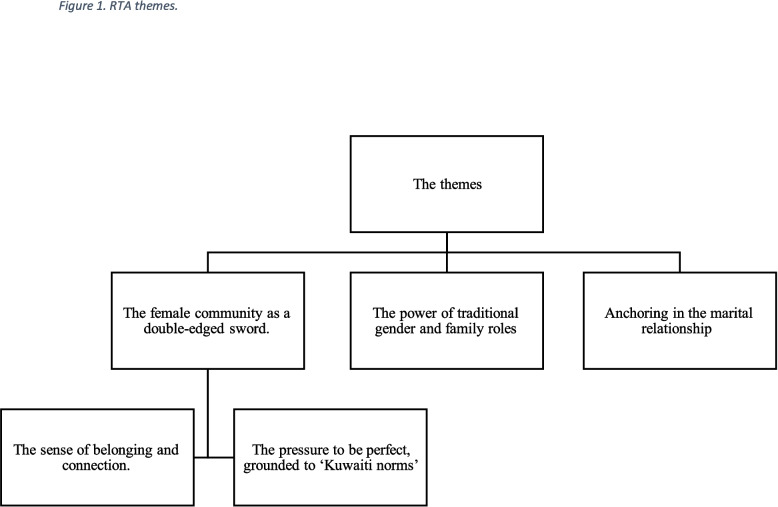
RTA themes

Three main themes and two subthemes interpreted from the data are shown in Fig. 1.

### Theme One: The female community as a double-edged sword

From participants’ perspective, the female community can provide substantial support, resources, and a sense of belonging and connection for women. On the other hand, they may also be judgmental and put more pressure on pregnant women.

### Subtheme One: The sense of belonging and connection

This subtheme explores the participants’ perceptions of their female community as supportive and positive, and playing a fundamental role in Kuwaiti women’s experiences during pregnancy. This community can provide a sense of connection and belonging and a place where women share their experiences and challenges, such as physical changes (e.g., weight gain), fatigue, or emotional changes (e.g., mood swings) encountered during pregnancy. For example, one participant said,* ‘‘Yeah, a friend I met at work, and we became close. It’s easy to open up to her and express all my feelings easily, I always end up feeling positive afterwards.’’ [P26].*

Women described listening to each other, expressing their feelings, and creating trust and emotional support that helped them coping with difficult circumstances. Another participant stated, *“When I had gestational diabetes, my mother-in-law had that, and she said it’s fine… All girls have gestational diabetes, in our society, of course, it comforted me a lot. When I think a lot, or when I obsess about things, she comforts me.’’ [P4]*

Female community members also played an important role in supporting a pregnant woman’s emotional health by exercising patience with her nervousness and mood swings: *“I became more nervous and sensitive. I’m trying to control myself. Whenever people around me see me in this state, they keep quiet and they disregard what I say because they know that I don’t have control over this, you know.” [P23]*

During the prenatal period, the surrounding female family units, such as in-laws and premarital family, can provide strong support to the women. Other participants also noted that in Kuwaiti society, irritable behaviour in pregnant women is often excused and met with understanding. This response aligns with cultural norms that emphasise compassion and support for expectant mothers.

Most participants mentioned the maid as the main source of help inside the house: *“Look I have a worker who helps me at home… thank God, so she is my number one help.” [P3]*. Another expressed,* “To be honest, I relied on her to cook before pregnancy… I also rely on her to cook because I won’t be able to cook, watch my daughter, and go to work all at once. I will not be able to do that.” [P25].*

Seeking additional help from family members can also reduce the burden of childcare. Most participants relied on their familial female networks; their sisters and mothers were the most frequent sources of help. For example, “*My mother and my sister visit me when I’m tired, they take my children. Sometimes I visit my sister’s house, and other times I visit my parents.” [P12].* Another participant said, *“I would send my sister a message asking if she is at home, and if I can drop my children over for a while. She tells me that I can drop them off, and that’s it. My sister also knows what my children eat and what they like, and so it is easier for me and for my children to go to her.” [P0].* Participants felt that familiarity with the children made the female family members better caregivers and gave the children a greater sense of security, while allowing mothers to feel more secure when they knew that their children were being taken care of by someone they trust.

### Subtheme Two: The pressure to be perfect, grounded to ‘Kuwaiti Norms’

Many of our participants perceived pressure from their female community to conform to ‘ideal’ expectations. This pressure could come from various sources, such as female family members, friends, or healthcare providers, and take the form of advice or judgments about a woman’s pregnancy choices or lifestyle.

One participant explained that *“My grandmother and those who are old, they are old-fashioned… They talk about herbs and nonsense. I don’t wish to ask them because I will not do anything they say (laughs), such as ‘Don’t put a leg over the other’. They don’t have medical proof, but you must play along so they don’t insist (laughs).” [P19].*

Other pregnant women expressed how the negative comments during their in-laws’ gatherings affected their mood; for example, ‘‘*It depressed me a little… Change in body weight, nose getting bigger and pigmentation. Society doesn’t leave one in peace. Once, I was at a women’s gathering at my in-law’s house, and a woman laughed and commented about my pigmentation… I can think well of her intention, but she cannot say that I turned dark in front of all the other women’’ [P4].*

The female community can be a breeding ground for harmful myths and misinformation regarding pregnancy and childbirth, as participants intimated. For instance, *“People scared me about breastfeeding my child while I’m pregnant. They say that the baby might feel tired or turn out short. When I read medical material, I found out that it is not true… I even asked the general practitioner and the midwife, and they said that there is no danger for the child or the baby.” [P25].*

Participants pointed out other myths that pregnant women should not exercise or lift things, or that a woman can bring on labor faster by drinking castor oil. Two participants referred to these beliefs as part of their culture; for instance, *“In our culture, a pregnant woman only walks, and people tell her to relax and not move or pressure herself when she exercises or walks, even if this thing is unlike her… For example, it’s unlike me to stay home for a long time, and they ask me to stay home and relax. When I relax at home, I become more exhausted. So, I must leave the house for fresh air” [P13].* To avoid these criticisms some participants were hiding their activities (e.g., secretly going to the gym).

One participant highlighted a crucial point about her mother’s reaction when she expressed her need to see a therapist; “*I used to vent to my mother and cry… I used to tell her that I’m depressed, and she used to leave me and tell me that I’m crazy. She’s afraid that I might have a file, and people would find out that I seek mental help” [P4].*

All the participants in this study asserted that they received unwanted suggestions from women in their community based on their pregnancy experience. Examples included: “*You should not do this or eat this”*; *“Don’t go to this doctor, I was going to die by her hands”; “Take a medical leave from work”; “Natural childbirth is better than C-sections, we all had natural birth”.* These suggestions and advice can be intrusive and unwelcome, and they can adversely affect mental health and well-being [17].

Several participants reported that, when they complained to their female network, their concerns were dismissed. For example,* “They tell me not to exaggerate, and that they all got pregnant, and nothing happened to them… I’m currently asking about breastfeeding, I attend classes, and if I talk about the subject, they tell me that I’m exaggerating, and that it’s easy and they all breastfed.” [P11].*

### Theme Two: The power of traditional gender and family roles

Theme two considers the power of traditional gender and family roles during the participants’ pregnancy experience.

*Traditional gender roles* in Kuwait shaped pregnancy experiences. One participant mentioned that *“He said that he doesn’t like to clean, and he doesn’t like me to tell him to enter the kitchen and cook. I once asked him to get me a cup of water, and he said that he doesn’t like to get water for women. He would never make me dinner or any meal, even if I’m tired, or if the children are very hungry. He would rather order from a restaurant rather than enter the kitchen.” [P10].*

On the other hand, other participants said that their husbands offered help with the household chores. However, they themselves felt the household work, especially cleaning the bathroom or general cleaning, was their responsibility.

Other participants mentioned that their husbands did not help them teach the children as the men could not tolerate doing that. For example, *“I will not find support regarding this matter at all (laughs). Forget about it… Rarely would you find a man who teaches the children. I don’t think he can tolerate opening a book.” [P2*].

Feelings of guilt emerged when women felt they were not meeting maternal expectations. For example, “*At the beginning (Silent)… What affected my mental state the most is the feeling of guilt that I didn’t give enough to my children.” [P12].* Another participant mentioned that she felt guilty during pregnancy as her husband cooked for them, and she usually left him without lunches or dinners—even though he was fine with that and never complained.

Societal pressure regarding fetus’s safety and gender also surfaced. For example, *“When I visited the doctor, she said that everything is fine, but the baby is positioned low, and that he must come up. I spoke to my mother, and she scolded me, she probably thought that it’s my fault, but it’s not.” [P27].* Another participant said that “*I’m alright with it, but ((pause)) my husband did not accept it. He wished for a boy… The reaction on his face affected me a lot a lot… It affected me to the extent that I didn’t attend my next appointment.” [P5].* She explained why her husband preferred a boy: *"It must be a boy, you know, the boy makes you proud. This causes much pressure on the pregnant woman."* Another example, *“It’s not up to me to decide whether I give birth to a girl or a boy. We entered 2022, and there is no difference between the boy and the girl… The society in general prefer boys, of course. The doctor told me that I may be pregnant with a girl, and when I told my grandmother about it, she hoped that the gender would change.” [P27].*

Age-related stigma was another concern for some participant. *“Honestly ((pause)) I was embarrassed for being pregnant in my 40 s, once a woman reaches her 40 s, she must stop giving birth. I’m so embarrassed, my nine-year-old boy and I were talking yesterday, and so, I told him, ‘I will have another baby’! He was like: you’re 40 and still thinking about kids?!” [P26].*

Kuwaiti men are stereotyped as not having strong feelings. Participants explained, "*You know how men don’t have very strong feelings. We, mothers, have stronger feelings. I wish there would be care… but I understand that the man’s personality is different from the woman’s. The woman has more emotions than the man so I don’t judge him.” [P4].*

The participants also mentioned different reasons that prevented men attending their wife’s appointments; for example, *“During the pregnancy follows ups with doctors, it is not acceptable for the man to be around his wife, my in-laws will even tell my husband he doesn’t have to attend my appointments, and they’ll criticize him” [P30].*

Traditional family roles could also intrude on maternal choices. For instance,” *Also, family traditions may affect what we’ll name the child. Honestly, I don’t have any problem accepting the names my husband choses, however because everyone is telling me that you will never get the chance to choose in the future… I had to argue with my husband about the idea that I’m the one who is carrying the weight and giving birth, and I don’t get the chance to choose!” [P30].*

On the other hand, in-laws may provide the pregnant woman with substantial emotional, information and practical support, as part of the traditional family roles in some families. Many participants mentioned that they received practical help and support from their families. For instance, *“My uncle (father in-law) helps me if there is anything missing in my house, or if something needs fixing. I call him, and he does things for me” [P8].* Another participant highlighted that, “*I don’t worry about my children if I want to go out, as I am living with in-laws. There are people at home all the time, thank God. I’m very lucky to be living at the in-law house.” [P17].*

For example, one participant highlighted that, *“Mom comes first, but now she’s alone, and all she does is think about our children, if they’re upset, or if we’re upset with our men… I try as much as I can not to tell her things. I used to talk to her before, and I would feel very comfortable talking to her.” [P1].*

Traditionally, women are expected to be polite, considerate, and deferent in social interactions with their elderly relatives. This can limit the support that they seek in some cases. For example, one participant highlighted that, *“Mom comes first, but now she’s alone, and all she does is think about our children, if they’re upset, or if we’re upset with our men… I try as much as I can not to tell her things. I used to talk to her before, and I would feel very comfortable talking to her.” [P1].* Explicitly, she explained that she was concerned that sharing her struggles with her mother would only add to her mother’s anxiety and worry. It was her belief that by keeping her worries to herself, she was protecting her mother from any stress and anxiety related to her own problems. Moreover, many participants revealed that they deal with intrusive questions from their elderly relatives by being patient and polite with them.

All the participants expressed that the premarital home is a good place to relax and receive care, with less formality than with in-laws. For example, “*I become comfortable when I attend my family’s gathering. My family is different. I wear whatever I like, and I go downstairs whenever I want. It’s different, and unlike the husband’s family… I will not lie down in front of my husband’s family, and I won’t feel comfortable.” [P15].* Many also highlighted that they enjoy family gatherings which provide them with attention, emotional closeness and support, and a sense of joy and belonging. For instance, *“You find them asking me to lift my legs and offering to massage my feet. My family is pampering me a lot. I can lift my legs and lie down and sleep around my family, so it’s fine.” [P16].*

### Theme Three: Anchoring in the marital relationship

The marital relationship can be seen as an anchor if it provides stability, security, love and support. Some women in the interviews were satisfied with what their husbands provided for them and seemed relaxed and less stressed. However, most complained about their husband not understanding their feelings, meeting their needs, or getting involved in childcare. Those pregnant women who complained focused on a lack of emotional support.

As stated by one participant, *“My husband helped me a lot. Sometimes I ask him to help me wear my shoes when I cannot wear them… He used to dress the children, wash them, and change their diapers. He helped me a lot.” [P7].* In another example, *“It’s giving me incentive, and it’s making me comfortable because he is considerate, and he carries the responsibility with me.” [P23].* Another participant explained that *“All my feelings are exposed, even when I’m angry at him, or when I laugh with him. He’s the closest person I talk to. Because we are friends more than we are husband and wife. I like to talk to him, and he likes to talk to me.” [P2].* Indeed, some participants perceived their husbands as understanding, caring and affectionate. For example, "*Of course, I express my anger at the moment, and then the opposite of anger happens… I cry (laughs). My husband understands my feelings at the same moment. He is considerate in this regard." [P11]*

However, most pregnant women in our study reported that their husband does not assist with childcare and household chores. For example, “*He’s not responsive or responsible at all. I would go to the doctor, the supermarket, and everywhere because he’s busy. He is not even cooperative. I do everything. I’m exhausted” [P1].*

Some participants stated they avoid talking to their husbands as their reactions fail to meet their needs or hurt them. For example, *“Sometimes I wish we can have a full conversation and connect, but once I see the look on his face that tells me he’s lost, I end the talk. I’m facing this a lot with pregnancy. I’m avoiding talking to him.” [P28].*

Participants often desired more emotional support from husbands than they currently received. For example, ‘‘*It’s true that everyone around me cares about me, such as my husband’s grandmother, my God grant her life, who really cares about me, but you would wish that the person you love cares about you.” [P4].*

Finally, some of our participants opined that many men do not realize the true extent of the physical and emotional challenges during pregnancy. For example, ‘‘*I would like to participate in this (research) so there would be more awareness amongst men regarding the pregnant woman’s suffering, the things that she thinks about, and what the hormones do to her.” [P18].*

## Discussion

This qualitative study offers insight into Kuwaiti women’s experiences of social support during pregnancy, especially in the context of cultural and traditional gender roles. It looks at an Asian population with high-income status, complementing other studies that focus on lower-income populations [61–64].

### Female communities: Supportive yet pressurising

Participants’ narratives confirmed that shared experiences within female networks fostered trust, emotional security, and resilience. Women can provide a listening ear to each other by expressing their feelings and thoughts through shared experiences. This can create a sense of trust, emotional support, and help women to cope with difficult circumstances. This type of connection can be beneficial during times of crisis or hardship, as it fosters a sense of self-worth and resilience, enabling them to face challenges more positively and actively [65, 66]. This is in line with the Self-Determination Theory (SDT) [67] which assumes that humans have an innate need to be connected to, and loved by, others. Connecting, attaching to, or relating to others can promote motivation, well-being, and satisfaction, whereas poor relationships can result in a low sense of self and motivation [68]. Sharing similar pregnancy experiences (e.g., gestational diabetes) providing an opportunity to normalise the experience, receive practical advice, and alleviate feelings of isolation and anxiety [69, 70]. Moreover, across Arab communities, both birth and death are typically met with significant community and family involvement. According to both Islamic and Arab cultural norms, these two events are occasions that require the support of extended family members and members of the wider community [30, 71]. The participants’’ expressions illustrate a housemaid’s important role in providing childcare and domestic services inside the house being one of the social norms for Arab Gulf families, and maids are often regarded as a status symbol [72–74]. Additionally, cultural expectations regarding gender roles and household duties may contribute to maid reliance [75]. For example, it is still widely accepted that the head of the house is a male and that the woman is primarily responsible for maintaining the household [40]. Accordingly, maids are seen as necessary to lighten the burden on the mother and conform to social expectations.

Yet these same networks also imposed high pressures. For example, female communities such as grandmothers often offered advice based on their life experiences, cultural beliefs, and wisdom, rather than scientific evidence. Such advice is thought to be passed down from generation to generation to protect and guide grandchildren [76, 77]. Moreover, negative comments about appearance and body changes during pregnancy leads these women to feel uncomfortable, unwelcome, or judged. They may also feel unappreciated and unaccepted, which may have a negative impact on their mental health and self-esteem [78]. Indeed, a systematic review of 22 studies showed that body dissatisfaction in pregnancy is associated with several factors, one of them being perceived socio-cultural pressure [79].

The enduring circulation of myths and unsolicited advice in female communities about exercise, breastfeeding, or labour induction, as reported in this sample, is also global. A recent systematic review of 25 studies from around the world which reported that myths and wrong beliefs regarding pregnancy and childbirth (e.g., eating behavior) persist not just in developing countries but also in developed countries [80]. This kind of unsolicited advice can be considered a form of social hindrance. Social hindrance refers to negative social interactions that hinder personal growth and well-being, which can harm an individual’s mental and emotional state [81].

Overall, the findings highlight the dual role of female communities, which provide different types of support but also pressure women to meet perfectionist standards and conform to expectations. However, this study uniquely identifies the dual hindrance of scrutiny and pressure from female communities, a concept also linked to female competition and social dominance [82]. Stockley and Bro-Jrgensen [82] found that female competition is centred around resource monopolisation and social dominance. Future research should continue to explore the complexities of how female communities can simultaneously provide understanding and support while imposing high expectations or pressure.

### Traditional gender and family roles

*Traditional gender roles* in Kuwait dictate that women care for the children and maintain the household. Indeed, in several cultures, men are not expected to help with childcare or housekeeping, limiting women’s support [38]. This is due to deeply ingrained beliefs regarding gender roles perpetuated by stereotypes and expectations. In the present research, many participants highlighted how traditional gender roles affected their pregnancy experience and perceived these roles as a hindrance to social support. Some husbands resist household tasks, likely due to men’s perception that they should not overtake traditional feminine responsibilities. This is because men are expected to be breadwinners and women homemakers [83]. In many societies around the world, chores such as cooking, cleaning, and laundry are still largely relegated to women. This may indicate that attitudes toward housework and gender distribution persist across generations as a cultural trait [84–87]. This is because women are traditionally viewed as primary carers in the home. Cultural norms and expectations often reinforce this [88]. Furthermore, societal norms and expectations reinforce the notion that men are not required to assume these responsibilities [89].

On the other hand, some participants did not have a problem with their husband’s absent roles inside the house, as they did not expect his help. From the cultural and discursive context, gender ideology is defined as the beliefs and values people hold about what is right for men and women [90, 91]. It defines how a particular society judges or evaluates a man’s or a woman’s behaviour. It is important to note that the language people use, their roles, and the behaviours they accept or reject reflect these attitudes and values. Accordingly, this gender ideology determines how people interact and communicate. Women and men, therefore, develop expectations of each other based on society’s expectations [92, 93].

Additionally, some participants indicated that men do not attend their wives’ appointments. In some cultures, men are prohibited from attending their wives’ gynaecological appointments because of stigma associated with men being involved in their partners’ healthcare. This is attributed to cultural beliefs regarding modesty and privacy [94]. Arab-Islamic culture views childbirth primarily as a woman’s venture, with minimal input from the husband and no expectation of active participation [95].

One of the factors that pressure pregnant women is gender preferences. Pressure to produce a male child—often justified by lineage concerns—echoes patterns documented across diverse societies [96, 97]. Another concern that arises from the interviews is age-related stigma toward late pregnancies. According to Lahad and Madsen [98], social clocks play a vital role in culturally scripting mothers aged 40 and older, and this perception is often based on patriarchal assumptions. As part of gender norms, women are expected to have children at an early age [99].

*Traditional family roles* in Kuwaiti society are complex and can vary depending on the individual family. For example, from participants experiences, these roles may allow in-laws and elderly relatives to be critical of the women’s pregnancy or motherhood choices (controlling, intrusive, even to the point of declaring that motherhood is not acceptable). Women may feel pressure to conform to outdated expectations. In Islam, showing respect and kindness to one’s elders is not only a moral obligation but also a way to obtain blessings [100]. The Holy Prophet said, ‘‘He who wishes that his sustenance be increased for him and his death day be delayed, then he should pay attention to his kinsfolk.’’ [101] (p. 89). Maintaining strong family ties is highly encouraged and considered a cornerstone of society. Moreover, in Islam, the family roles secure distinct rights within the family, and members are highly valued [100, 102].

During pregnancy, some women moved to their parents’ house for assistance. This kind of support enhances emotional security and well-being. Furthermore, knowing that family members are available to offer help and guidance when needed can provide great comfort and reassurance and a sense of belonging and acceptance, which can also enhance self-esteem and confidence [103, 104]. As a result, it may also act as a buffer against stress and difficult circumstances during life [105].

### Marital anchors and gaps

Emotional and practical partner support buffered stress, consistent with evidence that high-quality spousal involvement predicts better maternal well-being [106–113]. Participants revealed that having effective emotional and practical support creates a secure environment for them, helping them feel more relaxed and comfortable. The importance of emotional support from a husband is in line with several psychological theories about human innate needs. For example, to develop self-determination, a sense of belonging is essential, as is building close and affectionate relationships with others. These relationships and interactions can promote or thwart individual well-being [67]. A self-determination theory states that every human being has a need for competence, autonomy, and relatedness. It is defined as being connected, attached, or related to others [114]. It refers to the extent to which an individual thinks that others love them, support them, and care about their feelings and problems [115]. When those innate needs (e.g., relatedness) are not met, the individual feels distress, deep sadness, and dissatisfaction [67, 116]. This theory highlights husbands’ emotional support and relatedness may reduce wives’ stress, and vice versa.

Islam describes a marriage relationship filled with tranquility, care, and affection as a ‘sign of God’, which may foster social support between partners [100]. It also describes the relationship between husbands and wives as close, intimate, and protective: ‘‘They are like garments to you, and you are like garments to them’’ (2: p. 187). As a result, such relationships can strengthen women’s confidence and help them cope with parenting changes. It has been shown that having a supportive partner during pregnancy reduces stress and anxiety [110, 111]. Furthermore, it can make mothers feel more valued and secure [112]. Also, husbands’ involvement and support helps women feel less anxious about pregnancy, contributing to their positive outlook [110]. This can eventually benefit a woman’s mental health and emotional well-being [113].

Conversely, many women experienced emotional disconnection and communication barriers with their husbands, which they attributed to their husbands’ lack of understanding. This lack of understanding led them to refrain from expressing their needs and feelings. Esmaeilpour et al. [117] highlighted the significant role of communication skills in marital satisfaction. Similarly, a recent systematic review found that pregnant women often experienced a lack of emotional support from their husbands, describing them as less caring and unresponsive to the emotional changes they were undergoing [118, 119]. Additionally, most of the participants experienced a lack of practical help from their husbands. The issue of parental responsibility is common for many couples, particularly those with a traditional gender divide in parenting duties. In circumstances where one partner is not engaged, the other is left to carry the entire burden of childcare and other household responsibilities [110, 120]. As noted in the second theme, the lack of help may be due to traditional gender roles, resulting in men believing housework does not belong to them [85, 121].

### Strengths and limitations

Strengths of the present research include the use of qualitative methods that allowed for a comprehensive understanding of pregnant women’s perceptions and the use of WhatsApp interviews that encouraged frank and detailed disclosure. The study included a large sample of 31 pregnant women from various socio-demographic backgrounds throughout Kuwait.

However, the limitations of this study are that it was conducted during a transitional period of COVID-19 pandemic. It is possible the pandemic context influenced participants’ experiences. Another limitation was the use of WhatsApp calls to conduct the interviews. This may have led to a lack of non-verbal cues and personal connection [122, 123]. This could have impacted the depth and accuracy of the data collected.

### Implications

Our results highlight the importance of adapting social support measurements or interventions that are culturally sensitive. Most existing studies have used general measures or brief self-reports to assess social support during pregnancy. A single-item measure of social support (SIMSS) is often used in studies, asking participants to describe ‘‘who they turn to when they need assistance’’ [124]. Additionally, recent systematic reviews found the Oslo Social Support Scale (OSS-3) to be the most used tool [1, 2]. The scale consists of just three items that measure close confidants, feelings of concern, and relationships with neighbors with a focus on practical help. Although the OSSS-3 is considered feasible and valid, it cannot offer deeper details or comprehension of social support. This broad approach ignores the differences in how people from different cultures perceive and experience social support. According to Campos et al. [125], people’s perceptions of the social world are affected by cultural systems. For instance, looking at different populations living in the same geographical area revealed that Arabs find more emotional support from their family. In contrast, Jews find more support from their partner or close friend [33].

## Conclusion

This research addresses gaps in the literature, provides cultural insights into various experiences, and emphasises the importance of tailoring support according to the local context. It contributes to a better understanding of social support during pregnancy in the Gulf region and provides a basis for further investigation in this field. For example, following up on our qualitative research, we have taken up the themes that emerged in this study to further examine links between cultural orientation, positive and negative social experiences, and mental health and wellbeing in pregnancy, for women in Kuwait.

## Supplementary Information

Supplementary Material 1.

## Data Availability

The datasets used and/or analysed during the current study are available from the corresponding author on reasonable request.

## Abbreviations

RTA: Reflexive thematic analysis
SDT: Self-Determination Theory
SIMSS: Single-item measure of social support
OSS-3: Oslo Social Support Scale
